# The burden of inappropriate birth weight on neonatal survival in term newborns: a population-based study in a middle-income setting

**DOI:** 10.3389/fped.2023.1147496

**Published:** 2023-06-08

**Authors:** Tulio Konstantyner, Kelsy Catherina Nema Areco, Paulo Bandiera-Paiva, Ana Sílvia Scavacini Marinonio, Mandira Daripa Kawakami, Rita de Cássia Xavier Balda, Milton Harumi Miyoshi, Adriana Sanudo, Daniela Testoni Costa-Nobre, Rosa Maria Vieira de Freitas, Liliam Cristina Correia Morais, Monica La Porte Teixeira, Bernadette Cunha Waldvogel, Carlos Roberto Veiga Kiffer, Maria Fernanda Branco de Almeida, Ruth Guinsburg

**Affiliations:** ^1^ Departamento de Pediatria, Escola Paulista de Medicina, Universidade Federal de São Paulo, São Paulo, São Paulo, Brazil; ^2^ Departamento de Informática em Saúde, Escola Paulista de Medicina, Universidade Federal de São Paulo, São Paulo, São Paulo, Brazil; ^3^ Departamento de Medicina Preventiva, Escola Paulista de Medicina, Universidade Federal de São Paulo, São Paulo, São Paulo, Brazil; ^4^ Diretoria Adjunta de Produção e Análise de Dados, Fundação Sistema Estadual de Análise de Dados, São Paulo, São Paulo, Brazil; ^5^ Departamento de Medicina, Escola Paulista de Medicina, Universidade Federal de São Paulo, São Paulo, São Paulo, Brazil

**Keywords:** infant newborn, neonatal mortality, infant—small for gestational age, fetal macrosomia, developing countries, epidemiological studies

## Abstract

**Introduction:**

Premature birth, perinatal asphyxia, and infections are the main causes of neonatal death. Growth deviations at birth also affect neonatal survival according to week of gestation at birth, particularly in developing countries. The purpose of this study was to verify the association between inappropriate birth weight and neonatal death in term live births.

**Methods:**

This is an observational follow-up study with all term live births from 2004 to 2013 in Sao Paulo State, Brazil. Data were retrieved with the deterministic linkage of death and birth certificates. The definition of very small for gestational age (VSGA) and very large for gestational age (VLGA) used the 10th percentile of 37 weeks and the 90th percentile of 41 weeks + 6 days, respectively, based on the Intergrowth-21st. We measured the outcome in terms of time to death and the status of each subject (death or censorship) in the neonatal period (0–27 days). Survival functions were calculated using the Kaplan–Meier method stratified according to the adequacy of birth weight into three groups (normal, very small, or very large). We used multivariate Cox regression to adjust for proportional hazard ratios (HRs).

**Results:**

The neonatal death rate during the study period was 12.03/10,000 live births. We found 1.8% newborns with VSGA and 2.7% with VLGA. The adjusted analysis showed a significant increase in mortality risk for VSGA infants (HR = 4.25; 95% CI: 3.89–4.65), independent of sex, 1-min Apgar score, and five maternal factors.

**Discussion:**

The risk of neonatal death in full-term live births was approximately four times greater in those with birth weight restriction. The development of strategies to control the factors that determine fetal growth restriction through planned and structured prenatal care can substantially reduce the risk of neonatal death in full-term live births, especially in developing countries such as Brazil.

## Introduction

Although a reduction in the number of neonatal deaths has occurred in recent decades, mainly related to the greatest availability of technical and clinical resources, many newborns continue to die worldwide (1). Globally, newborn deaths still account for 47% of under-5 deaths, and 2.4 million children died in their first month of life in 2019, with approximately 6,700 neonatal deaths every day (2).

The risk of neonatal death is closely associated with three preventable and treatable conditions: complications of prematurity, intrapartum-related neonatal deaths (including birth asphyxia), and neonatal infections (1). Other associated factors are congenital anomalies, nutritional status at birth (mainly fetal growth restriction), and health-related factors, such as income, educational attainment, fertility rate, housing, and access to healthcare (1, 3, 4).

Adequate intrauterine growth has been the focus of many studies that identified changes in the growth of the embryo/fetus associated with neonatal death and clinical/metabolic outcomes at all stages of life. The nutritional classification at birth has been used as a marker for morbidity and mortality in newborns and in all pediatric age groups (4).

On the one hand, newborns small for gestational age (SGA), especially severe SGA, are at greater risk of neonatal death than those who do not show signs of intrauterine growth restriction. On the other hand, newborns large for gestational age (LGA) have a higher frequency of morbidities such as brachial plexus and skeletal injuries, hypoglycemia, and perinatal complications. When present, these morbidities may lead to complications that contribute to neonatal death, such as asphyxia in term births (4–6).

Neonatal deaths associated with inappropriate birth weight differ according to gestational age, the method used to classify birth weight adequacy, and the ethnic and racial characteristics of the population studied (5). The risk of clinical complications and death is substantially higher in preterm infants (4). The effect of inappropriate birth weight is potentially overshadowed in premature infants by the intensity of determination of the functional immaturity of these newborns on the risk of death (4, 5).

There are few population-based studies that assess the association of these growth deviations at birth with neonatal death, specifically in term newborns, who do not suffer the clinical consequences of the functional immaturity of preterm newborns and potentially have the greatest chances of surviving the neonatal period (4–6).

## Materials and methods

The aim of the study was to verify the association between inappropriate birth weight and neonatal death in term live births.

This is an observational follow-up study during the neonatal period (0–27 days of life), including all term live births with gestational age ≥37 weeks and <42 weeks from 2004 to 2013 from mothers residing in the State of Sao Paulo, Southeast Brazil. Sao Paulo is the richest and most populous Brazilian state, with 45,289,733 inhabitants as on January 16, 2023 (approximately 20.8% of the Brazilian population) (7).

Data were collected from a database built by the deterministic linkage of death and birth certificates after taking steps to ensure the suitability for use in quantitative scientific research (8). Those secondary health data were provided by the SEADE Foundation, the institution officially responsible for managing birth and death records in the State of São Paulo. The civil registry of births and deaths covers 99.7% and 99.8% of these events in the state (9). The details of that procedure have been reported elsewhere (10).

Infants without birth weight data, those with congenital anomalies, and twins and neonates born outside the hospital were excluded. Moreover, newborns with birth weight *z*-score values lower than −3 for 37 weeks and 0 days of gestation (1,640 g for girls and 1,590 g for boys) and greater than 3 for 41 weeks and 6 days of gestation (4,910 g for girls and 5,080 g for boys) were considered subjects with values that differed significantly from other observations (outliers) and, therefore, were removed from the sample (11).

The following data were collected from the birth certificates: maternal age, marital status and maternal education, number of previous pregnancies, number of prenatal visits, type of delivery, time and date of birth, sex of the newborn, 1st and 5th minute Apgar score, birth weight, and gestational age range. From death certificates, date and the age of death were retrieved.

In the Sao Paulo State, birth records provide information on gestational age by group, not week and days. Term gestation was considered when the range between 37 and 41 weeks was reported on the birth certificate. Therefore, we classified the birth weight adequacy based on this characteristic of the database as very small, normal, or very large. To define very small for gestational age (VSGA), values lower than the 10th percentile of birth weight for 37 weeks (2,330 g for girls and 2,380 g for boys) were considered. To define very large for gestational age (VLGA), values higher than the 90th percentile of birth weight for 41 weeks and 6 days (4,000 g for girls and 4,160 g for boys) were considered. These values were based on the Intergrowth-21st birth weight tables (11).

Neonatal death (death 0–27 days after birth) was defined as the main outcome of the study, which was measured as time to death (in days) during the neonatal period (0–27 days) and the status of each subject (death or censorship) until the end of the observation period.

Categorical variables are presented as the relative frequency according to birth weight adequacy. Survival functions were calculated and displayed using the Kaplan–Meier curve according to the birth weight for gestational age (very small, normal, or very large). We used the log-rank test for equality of survivor functions. Univariate and multivariate survival analyses using Cox regression were performed to investigate neonatal death-associated factors. Univariate associations with *p*-values under 0.20 led variables to be eligible to enter multiple model analysis.

The annual trend of the neonatal mortality rate was assessed by Prais–Winsten generalized linear regression through the calculation of the annual percentage change (APC). In addition, the interaction between birth weight adequacy and year of birth associated with neonatal death was estimated in the adjusted Cox regression analysis.

All statistical analyses were performed using Statistical Package Stata/SE 14.2 (Stata Corp LLC, College Station, TX, United States). A maximum level of 5% (*p*-value equal to 0.05) was chosen to indicate a statistically significant association.

The study was approved by the Ethics Committee on Human Research of Escola Paulista de Medicina—Universidade Federal de São Paulo (# 2,580,929) and by the Board of Directors of SEADE Foundation. There was no direct contact with the study subjects or any personal identification. The dataset was accessed from October 2018 to June 2021, and confidentiality of information was fully preserved before access.

## Results

From 2004 to 2013, there were 6,059,454 live births in the São Paulo State, Brazil. Of these, 5,860,736 (96.7%) had known gestational age. Of these, 575,441 (9.8%) were preterm births. Among the 5,285,295 identified term births, 143,614 were excluded because of one or more exclusion criteria. The other 399,279 subjects were excluded only from the multivariate analysis due to the absence of data on the selected variables to compose the final logistic model. Therefore, a total of 5,141,681 term live births were selected and studied in the univariate/bivariate analysis and 4,742,402 in the multivariate analysis, amounting to a sample loss of 2.7% and 7.6%, respectively.

There were 6,186 term infant deaths in the first 27 days of life (0.12%), with a neonatal mortality rate of 12.03 for 10,000 live births during the period of 2004 to 2013. The prevalence rates of VSGA and VLGA were 1.8% (*n* = 90,822) and 2.7% (*n* = 141,041), respectively. Table 1 shows the characteristics of the study population by group of birth weight adequacy.

**Table 1:** Maternal and infant characteristics according to birth weight adequacy (São Paulo State, Brazil, 2004–2013).

		Birth weight adequacy		
		VSGA	NGA	VLGA
Maternal characteristics				
Prenatal visits	No	2.7	0.8	0.7
	1–3	6.2	3.2	2.6
	4–6	23.9	17.4	16.3
	≥7	67.2	78.6	80.4
Cesarean section	Yes	53.1	56.5	68.0
Age (years)	<20	18.7	15.7	8.8
	≥35	14.4	12.1	16.3
	20–34	67.0	72.3	74.9
Education (years)	<8	32.2	24.4	28.6
	8–11	53.3	56.5	56.0
	≥12	14.5	19.1	15.4
Married	No	59.8	52.0	50.1
Multiparity	Yes	54.0	57.8	71.3
Infants’ characteristics				
Sex	Male	48.4	51.1	49.6
Birth weight	<2,500 g	100.0	2.0	0.0
1st minute Apgar	<7	9.4	5.2	7.8
5th minute Apgar	<7	1.4	0.6	0.8

VSGA, very small for gestational age; NGA, normal for gestational age; VLGA, very large for gestational age.

Data are displayed as percentages.

**Figure 1:**
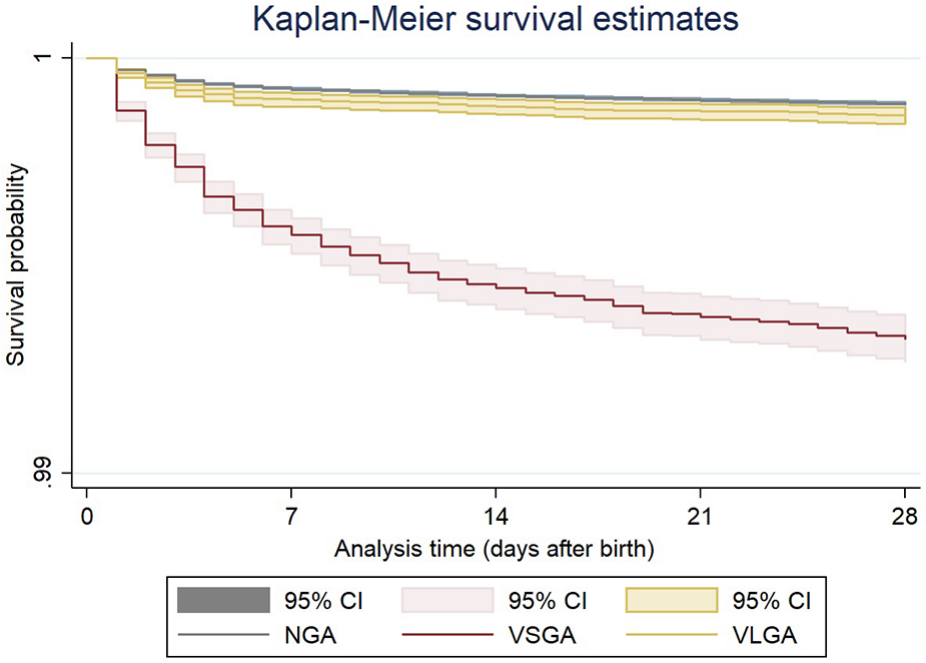
Kaplan–Meier survival estimates according to the three groups of birth weight adequacy of term live births, Sao Paulo State, Brazil, 2004–2013. Log-rank test: *p* < 0.001. NGA, normal for gestational age; VSGA, very small for gestational age; VLGA, very large for gestational age.

Figure 1 shows a significant difference in the risk of neonatal death between the three groups of birth weight adequacy (log-rank, *p* < 0.001). Newborns with both VSGA [hazard ratio (HR) = 6.19; 95% CI: 5.69–6.72] and VLGA (HR = 1.26; 95% CI: 1.09–1.45) had a higher probability of not surviving the neonatal period.

**Figure 2:**
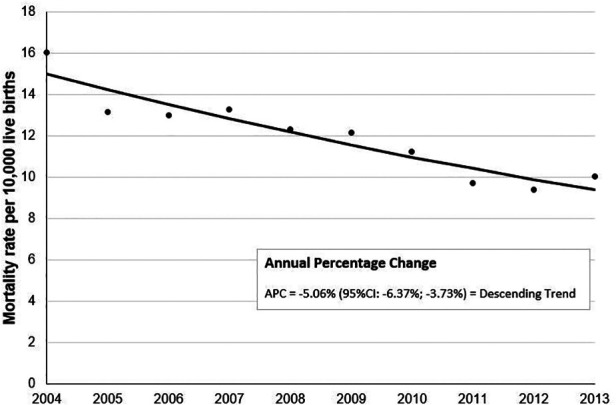
Annual percentage change of standardized mortality rates per 10,000 term live births, São Paulo state, Brazil, 2004–2013.

Additionally, the year of birth was associated with neonatal death. Standardized mortality rates showed a descending trend in the analyzed period, from 14.99 per 10,000 live births in 2004 to 9.39 per 10,000 live births in 2013 (APC = −5.06; 95% CI: −6.37 to −3.73) (Figure 2).

The adjusted Cox regression analysis showed a significant increase in mortality risk for VSGA infants (HR = 4.25; 95% CI: 3.89–4.65; *p* < 0.001). However, VLGA did not remain significantly associated with neonatal death in the multiple Cox regression model. Seven other variables were associated with the risk of dying during the neonatal period, regardless of the year of birth: male sex, 1st minute Apgar score <7, <7 prenatal visits, single mothers, multiparity, maternal age <20 years, and less education time (Table 2).

**Table 2:** Hazard ratios of variables associated with neonatal death in term live births (São Paulo State, Brazil, 2004–2013).

		Crude analysis		Multiple analysis	
		HR (95% CI)	*p*-value*	HR (95% CI)	*p*-value*
Maternal characteristics					
Prenatal visits	No	3.93 (3.40–4.58)	<0.001	2.52 (2.15–2.97)	<0.001
	1–3	2.25 (2.02–2.49)	<0.001	1.69 (1.51–1.89)	<0.001
	4–6	1.48 (1.39–1.57)	<0.001	1.18 (1.11–1.27)	<0.001
	≥7	1.00		1.00	
Cesarean section	Yes	0.99 (0.94–1.04)	0.584	—	—
	No	1.00		—	—
Age (years)	<20	1.35 (1.27–1.44)	<0.001	1.09 (1.01–1.17)	0.030
	≥35	1.14 (1.06–1.23)	0.001	1.04 (0.96–1.13)	0.375
	20–34	1.00		1.00	
Education (years)	<8	2.58 (2.36–2.81)	<0.001	1.71 (1.55–1.88)	<0.001
	8–11	1.66 (1.52–1.80)	<0.001	1.30 (1.18–1.42)	<0.001
	≥12	1.00		1.00	
Married	No	1.42 (1.35–1.49)	<0.001	1.12 (1.06–1.19)	<0.001
	Yes	1.00		1.00	
Multiparity	Yes	1.09 (1.04–1.15)	0.001	1.15 (1.09–1.23)	<0.001
	No	1.00		1.00	
Infant's characteristics					
Birth weight	VSGA	6.19 (5.69–6.73)	<0.001	4.25 (3.89–4.65)	<0.001
Adequacy	VLGA	1.26 (1.09–1.45)	0.002	0.99 (0.85–1.16)	0.948
	NGA	1.00		1.00	
Sex	Male	1.31 (1.24–1.38)	<0.001	1.21 (1.15–1.28)	<0.001
	Female	1.00		1.00	
1st minute Apgar	<7	17.34 (16.49–18.24)	<0.001	15.72 (14.90–16.59)	<0.001
	≥7	1.00		1.00	

VSGA, very small for gestational age; NGA, normal for gestational age; VLGA, very large for gestational age; CI, confidence interval; HR, hazard ratio.

* Cox regression.

Multiple model: adjusted for year of birth (*n* = 4,742,402).

Additionally, in the adjusted Cox regression analysis, there was no interaction between birth weight adequacy and year of birth associated with neonatal death (*p* = 0.970), showing that the risk of neonatal death for both VSGA and VLGA did not vary significantly over the years.

## Discussion

Newborn survival was not specifically addressed in the Millennium Development Goal (MDG) framework and consequently received less attention and investment (12). According to the “Every Newborn” action plan, now is the time for the global health community to prioritize the prevention of newborn deaths, and by 2035, all countries should reach the target of 10 or fewer newborn deaths per 1,000 live births and continue to reduce death and disability, ensuring that no newborn is left behind (1).

The present study shows a neonatal mortality rate in the state of Sao Paulo from 2004 to 2013 of 12.03 for 10,000 live-term births, which represents 15% of the neonatal mortality rate identified in all newborns in the same period and region (80.0 for 10,000 live births) (10). There was a decrease in the risk of neonatal death over the 10-year period, as shown in a study that evaluated all live newborns (10). This similarity suggests that the reasons that led to the decrease in these rates in the State of São Paulo reached all gestational ages globally.

Additionally, we found an increased and stabilized risk of neonatal death for VSGA during the years but not for VLGA term infants. Seven other factors associated with death during the neonatal period were found. This evidence reinforces the multifactorial nature of neonatal death, including important social and economic factors that are unevenly distributed across the state, as commonly seen in middle-income countries (13).

It is important to comprehensively understand the occurrence of neonatal deaths, considering the temporal sequence and the interrelation of multiple determining variables, including community-level contextual data and socioeconomic and proximal determinants, covering maternal, neonatal, prenatal, delivery, and postnatal characteristics (14).

Furthermore, it is not always possible to distinguish causality from association when one phenomenon is statistically linked to another (15). Even if basic inferential criteria are met, health studies are increasingly finding association networks to explain multifactorial events such as neonatal death (16). Consequently, the analysis of neonatal deaths requires not only the use of sophisticated epidemiological and statistical approaches that need special computing tools/software but also critical and cautious interpretations of the results, especially with regard to biological plausibility and the intensity of the estimated effect measures (17).

Specifically, SGA has been associated with a higher neonatal death risk in low-income and middle-income countries, as we found in the most populous Brazilian state (4, 17, 18). This finding shows that the presence of intrauterine growth restriction constitutes a risk factor for neonatal death (4, 18, 19). Additionally, SGA newborns have a considerably higher risk of morbidity, such as neonatal infections, perinatal respiratory depression, jaundice, polycythemia, hypoglycemia, poor feeding, and hypothermia. These clinical conditions, in turn, make them more likely to die in the neonatal period and beyond. In fact, The Child Health Epidemiology Reference Group (CHERG) showed that being SGA is associated with an increased risk of neonatal and post-neonatal mortality compared with infants who are normal for gestational age (NGA) (4).

However, in contrast to our findings, SGA is not always associated with or consistently predicts neonatal death (20). It is possible that being SGA does not always reflect gestational health problems. SGA at the 10th percentile was originally equated with a clinical syndrome of fetuses damaged by poor growth *in utero*. Although this syndrome does not strictly correspond to the clinical features of fetal growth restriction, it is more often referred to today (21). In general, there are multifactorial characteristics that lead the newborn to have a lower birth weight than their reference population pairs, including genetic inheritance (18, 22).

**Figure 3:**
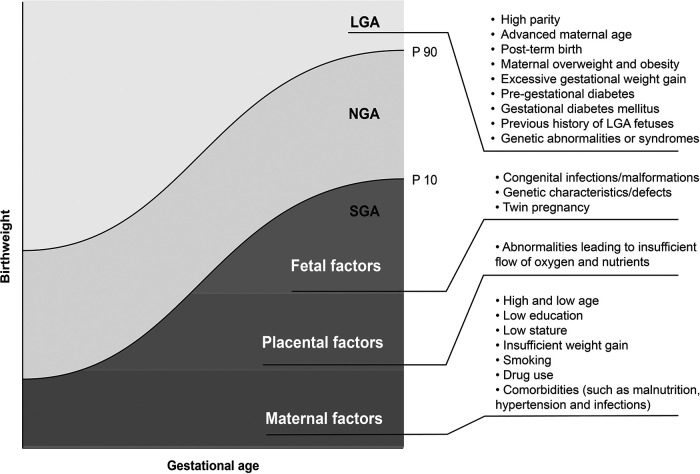
Risk factors for LGA (cutoff point: 90th percentile) and SGA newborns (cutoff point: 10th percentile). LGA, large for gestational age; NGA, normal for gestational age; SGA, small for gestational age.

Interestingly, restricted and excessive fetal growth are also multifactorial events like neonatal death itself. Risk factors for LGA mainly include maternal and gestational factors. In turn, risk factors for SGA include fetal, placental, and maternal factors (Figure 3) (4, 18–23).

While SGA is not itself a pathological category, the high risks among those at the lowest weights for gestational age may represent unknown causes and pathological processes (24). Even though this nutritional deviation at birth may be associated with other clinical conditions, which most directly lead to neonatal death, child health professionals must be aware of its occurrence to perform prevention actions of this outcome (23, 24).

In fact, neonatal death prevention strategies are interlinked with actions to prevent restricted fetal growth, avoid maternal malnutrition, and control gestational hypertension. Acting before birth, or even before conception, seems to be the most effective strategy to prevent clinical and metabolic conditions that trigger pathophysiological processes that determine fetal growth restriction and lead to a higher risk of neonatal death. When considering stillbirths not studied here, SGA has been repeatedly associated with this undesirable outcome, and our methodological strategy may thus have underestimated the effects of restricted fetal growth on human survival from conception (25).

In middle-income countries, in general, such as Brazil, the focus of the public health system during the last two decades has been mainly directed toward the development of infrastructure for the hospitalization of sick newborns at the expense of the quality of care in primary care, which includes prenatal care (26). Therefore, now seems to be the time to further improve the quality of prenatal care, which potentially prevents SGA and other determinants of neonatal death (27, 28).

Thereby, health managers must direct their attention and allocate available resources to strategies for controlling and preventing neonatal death. The noble objective of saving newborn lives should be planned in advance by removing risk factors, which is potentially the most effective way to reduce the mortality rate and promote child health, especially in developing countries such as Brazil. This improvement requires trained and equipped health professionals to work in primary healthcare and the availability of essential services and resource infrastructure. Efficient planning of actions within the existing sociocultural context, involving all levels of prevention, must be the basis of national public policies (27, 28).

With regard to our findings, developing strategies to control factors that determine SGA birth (fetal, placental, and maternal factors) can substantially reduce the risk of neonatal death. Despite this, it should be noted that our results should be carefully compared with other studies, as SGA is variously defined as the lowest 10th, 5th, 3rd, or 2.5th percentiles of birth weight according to gestational age (29). Moreover, the way that we defined inappropriate birth weight was based on week ranges, which may have led some SGA and LGA newborns to be classified as normal. Thus, we work with birth weight deviations, but mainly with the extremes. Although our study was carried out in a population-based sample, this may have led to under- or overestimation of the associations. Therefore, the comparison of our results in other methodological contexts should be considered with caution.

The complex nature of neonatal death determinants motivated the multivariate statistical analyses to independently identify the association between anthropometric nutritional status at birth and this outcome in term live births. Therefore, the findings gain greater validity and provide the broad perspective necessary to understand events that are triggered by multiple factors (30). However, because the analysis was based on an existing dataset, we were limited to the use of variables found in the death and birth certificates. For instance, our study did not evaluate the effect of neonatal clinical conditions and hospital assistance resources, which several studies have considered important neonatal death determinants.

On the other hand, São Paulo is the only Brazilian state that developed, over decades, its own system of producing independent vital statistics, which manages to relate continuous data from the civil registry with epidemiological data originating from death and live birth certificates, producing comprehensive information that allows a consistent analysis of child health indicators (31).

In this context, the risk of neonatal death in full-term live births was approximately four times greater in those with birth weight restriction in the São Paulo State. We did not identify VLGA as a risk factor for neonatal death. Despite the need to take some precautions, the findings of the present study can be generalized to areas or countries with socioeconomic characteristics such as those of the State of São Paulo, a middle-income setting.

Once countries are advised to prioritize child survival policy and programs based on their child cause-of-death composition, our findings may guide prevention and management strategies in line with the Sustainable Development Goals target for child survival (32). The prevention of fetal growth restriction through planned and structured prenatal care with balanced dietary guidance, recommendation not to smoke, maternal disease control, and gestational regular weight gain potentially contributes not only to reducing early death in full-term newborns but also to reducing its consequences and the global burden attributable to this suboptimal fetal growth (19).

Additional evaluations of population-based databases in different socioeconomic contexts and those that include clinical characteristics of the neonatal period should be carried out to confirm the findings of this study.

## Data Availability

The raw data supporting the conclusions of this article will be made available by the authors, without undue reservation.
